# Why Do Birds Flock? A Role for Opioids in the Reinforcement of Gregarious Social Interactions

**DOI:** 10.3389/fphys.2019.00421

**Published:** 2019-04-12

**Authors:** Lauren V. Riters, Cynthia A. Kelm-Nelson, Jeremy A. Spool

**Affiliations:** ^1^Department of Integrative Biology, University of Wisconsin–Madison, Madison, WI, United States; ^2^Division of Otolaryngology-Head & Neck Surgery, Department of Surgery, University of Wisconsin–Madison, Madison, WI, United States; ^3^Department of Psychological and Brain Sciences, University of Massachusetts Amherst, Amherst, MA, United States

**Keywords:** mu opioid receptors, reinforcement, social cohesion, affiliation, songbirds, medial preoptic area, ventral tegmental area, periaqueductal gray

## Abstract

The formation of social groups provides safety and opportunities for individuals to develop and practice important social skills. However, joining a social group does not result in any form of obvious, immediate reinforcement (e.g., it does not result in immediate copulation or a food reward), and individuals often remain in social groups despite agonistic responses from conspecifics. Much is known about neural and endocrine mechanisms underlying the motivation to perform mate- or offspring-directed behaviors. In contrast, relatively little is known about mechanisms underlying affiliative behaviors outside of these primary reproductive contexts. Studies on flocking behavior in songbirds are beginning to fill this knowledge gap. Here we review behavioral evidence that supports the hypothesis that non-sexual affiliative, flocking behaviors are both (1) rewarded by positive social interactions with conspecifics, and (2) reinforced because affiliative contact reduces a negative affective state caused by social isolation. We provide evidence from studies in European starlings, *Sturnus vulgaris*, that mu opioid receptors in the medial preoptic nucleus (mPOA) play a central role in both reward and the reduction of a negative affective state induced by social interactions in flocks, and discuss potential roles for nonapeptide/opioid interactions and steroid hormones. Finally, we develop the case that non-sexual affiliative social behaviors may be modified by two complementary output pathways from mPOA, with a projection from mPOA to the periaqueductal gray integrating information during social interactions that reduces negative affect and a projection from mPOA to the ventral tegmental area integrating information leading to social approach and reward.

Many well-studied social behaviors have primary reproductive or survival functions. For example, courtship behaviors are used to attract mates, agonistic behaviors are used to defend breeding and feeding territories, and food-begging calls are used to acquire food. These behaviors are directed toward a specific goal and can be reinforced by immediate, observable outcomes. For example, courtship can be rewarded by copulation, agonistic behaviors can be reinforced by immediate departure of a rival, and food-begging can be rewarded by receipt of food. However, outside these primary contexts, animals engage in several behaviors for which immediate functions and reinforcing factors are difficult to determine. This includes the formation and maintenance of social groups in gregarious animals.

The formation of social groups has adaptive benefits (e.g., safety and improved foraging efficiency; Powell, 1974; Lazarus, 1979; Sullivan, 1984; Thiollay and Jullien, 1998), and interactions within groups allow animals to develop and practice important social skills that can be used later in goal-directed contexts (Himmler et al., 2013; Vanderschuren and Trezza, 2014; Pellis and Pellis, 2017; Riters et al., 2017). However, joining a group does not result in any form of obvious, immediate reinforcement (e.g., it does not result in copulation or a food reward), and animals at times will remain in social groups even in the face of agonistic interactions with conspecifics. The formation and maintenance of cohesive social groups has evolved several times in vertebrate lineages, suggesting that gregariousness is not only adaptive, but that social grouping is reinforced by some external or internal mechanism at the level of individuals. Much is known about neural and endocrine mechanisms that reinforce mate-, rival-, and offspring-directed behaviors. In contrast, relatively little is known about mechanisms underlying the motivation for animals to affiliate in non-sexual social groups outside of these primary, reproductive contexts. Here, we review studies on flocking in non-reproductive contexts in songbirds that are beginning to fill this knowledge gap.

## Introduction to Non-Reproductive Flocking Behavior

When not mating or defending territories, many animals are solitary; however, there are notable exceptions, with birds ranking as among the most gregarious vertebrates. Birds are well-known for their remarkable flocking behavior. Members of some species spend most of their lives surrounded by flock mates (Goodson and Kingsbury, 2011), while others display predictable seasonal changes in sociality. For example, many birds shift seasonally from pair or solitary living during the breeding season to the formation of flocks for migration or overwintering after the breeding season (e.g., Emlen, 1952a; Feare, 1984; Eens, 1997; Wilson et al., 2016). The factors promoting gregariousness in birds have long been a source of interest. Early ethologists suggested that flocking can result either from non-social factors (e.g., individuals attracted to a common resource such as shade or a food patch) or social factors (i.e., mutual conspecific attraction or an aversion to being alone), with each of these factors likely at play in most flocks (Emlen, 1952a).

The proposed social influences suggest that flocks may result from the integration of both positive reinforcement (i.e., a behavior is strengthened because it leads to a positive outcome) and negative reinforcement (i.e., a behavior is strengthened because it leads to reduction of an aversive state), such that flocking behavior is strengthened because interactions with flock mates both (1) induce a rewarding, positive affective state and (2) reduce a negative affective state caused by social exclusion or isolation, thus creating a complementary system (i.e., positive reinforcement from affiliative interactions and negative reinforcement from termination of isolation).

### Flocking Behavior and Positive Reinforcement

**FIGURE 1:**
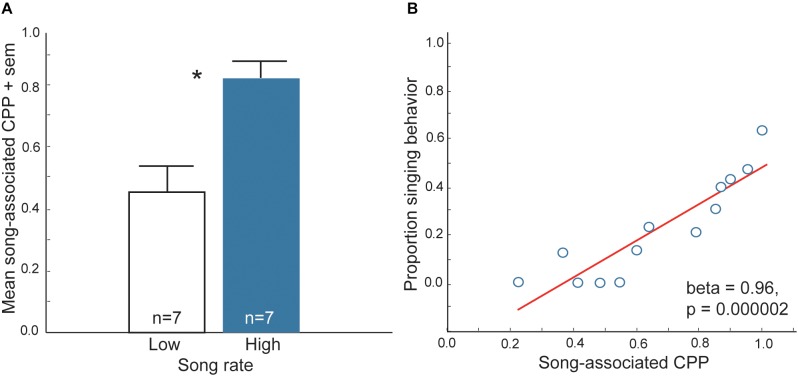
Evidence that vocal-social interactions in starling flocks are associated with a positive affective state. **(A)** Mean time spent on the side of a CPP apparatus that had been paired previously with either low (open bar) or high (filled bar) rates of singing behavior in flocks of male starlings. ^∗^*p* < 0.05. **(B)** Correlation between song and CPP in male starlings. *Y*-axis represents the proportion of all vocal behaviors that were songs produced by males during and just prior to being placed in one side of the CPP apparatus (song-paired side). The *X*-axis represents the proportion of time males spent on the previously song-paired side of the apparatus the following day (CPP, considered a reflection of song-associated reward). Each point represents data from a single male. Figures redrawn from Riters and Stevenson (2012).

At least three lines of research support the proposal that flocking is strengthened by mutual positive associations with flock mates. First, in choice tests zebra finches, *Taeniopygia guttata* prefer to spend more time near larger flocks of conspecifics (consisting of 10 same-sex individuals) compared to smaller flocks (consisting of two same-sex individuals) (Kelly et al., 2011). This finding indicates that in a species that is highly gregarious in nature, the presence of large numbers of conspecifics may be rewarding. Second, isolated European starlings, *Sturnus vulgaris* are willing to work (i.e., trigger sensors) to view images of conspecifics in the absence of any other reward. Furthermore, they respond more to pictures of starlings compared to pictures of landscapes or monkeys (Perret et al., 2015). This demonstrates that conspecific, social stimuli are primary reinforcers in this social species. Third, studies using conditioned place preference (CPP) tests [a common method to assay reward (Carr et al., 1989; Tzschentke, 2007; Trezza et al., 2009; Riters et al., 2013)] show that vocal-social interactions in non-breeding flocks of starlings and zebra finches are associated with a positive affective state (Riters and Stevenson, 2012; Riters et al., 2014; Hahn et al., 2017). In these studies, flocks of male starlings and zebra finches were observed singing in aviaries for 30 min. Each bird was then immediately placed individually into one of two distinctly decorated sides of a conditioning cage for 30 min and afterwards returned to its home aviary. The next day each bird was placed back into the conditioning cage and allowed to move freely between the previously song-paired and the non-song-paired sides of the cage, and the amount of time each individual spent on each side was recorded. The prediction was that if vocal-social interactions in non-breeding flocks are associated with a positive affective state, then birds would learn to associate the positive affective state with the distinctly decorated side of the cage and when given a choice spend most of their time on that side (i.e., they would develop a CPP). Results demonstrated that both male zebra finches and starlings developed a CPP for the chamber that had been paired with production of song in gregarious flocks (Riters and Stevenson, 2012; Riters et al., 2014; Hahn et al., 2017) (Figure 1). However, birds did not develop a CPP associated with song produced in mating or agonistic contexts, indicating that the factors that reward goal-directed, immediately reinforced behaviors differ from those underlying affiliative behaviors produced in non-sexual, gregarious contexts.

Although CPP tests are commonly used to examine rewarding properties of drug use, feeding and sexual behaviors, the function of CPPs in wild animals in natural contexts is seldom considered. The finding that vocal-social interactions in gregarious flocks can lead to the development of a CPP suggests that a natural function of this type of conditioning may be to strengthen group cohesion through a conditioned preference for a particular flock. Overall, these findings offer support for the hypothesis that social interactions in gregarious contexts can be positively reinforced.

### Flocking Behavior and Negative Reinforcement

It is common to observe birds that are separated from flocks appearing to rush to reunite with conspecifics. Early ethologists compared the motivation to flock in gregarious animals to a “hunger, a craving or sensation of discomfort” or a “state of agitation” in the absence of a physical requirement that can only be relieved by reunion with a flock (Trotter, 1916; Craig, 1918; Emlen, 1952a). This suggests that flocking may be strengthened not only because interactions with flock mates induce a positive affective state but also because they reduce a negative affective state. That is, flocking may be negatively reinforced. In gregarious vertebrates, social separation is painful (Macdonald and Leary, 2005; Eisenberger, 2012). For example, separation of young rats from mothers or the removal of guinea pigs, domestic chicks or zebra finches from conspecifics leads to the production of “distress vocalizations” or contact calls that are eliminated by reunion with group mates (Zann, 1985; Hofer et al., 1993; Kyuhou and Gemba, 1998; Warnick et al., 2005). Several studies demonstrate that the same neural systems and modulators that process physical pain also regulate the pain of social separation, and this is proposed to be adaptive given that social disconnection in gregarious species threatens survival (Macdonald and Leary, 2005; Eisenberger, 2012). For example, treatments that reduce physical pain (i.e., induce analgesia) are also found to reduce signs of social pain in animals as well as activity in brain regions underlying physical pain. This includes treatment with opioids such as morphine and even treatment with the common pain reliever acetaminophen (Herman and Panksepp, 1978; Panksepp et al., 1978; Dewall et al., 2010).

The data showing that social and physical pain share underlying mechanisms lead to the prediction that social interactions in flocks may reduce social pain. Some support for this idea comes from a CPP study in male zebra finches. In zebra finches, separation from a flock increased production of distance contact calls, which are triggered by separation from partners or flock mates and reduced when individuals are reunited (Guttinger and Nicolai, 1973; Simpson and Vicario, 1990; Zann, 1996). In contrast to the CPP identified for song in flocks described above, zebra finch contact calls correlated negatively with an individual’s affective state measured using CPP (i.e., males that produced high numbers of contact calls avoided the side of the CPP chamber associated with these calls) (Riters and Stevenson, 2012). This indicates that the affective state associated with separation-induced calls is negative.

**FIGURE 2:**
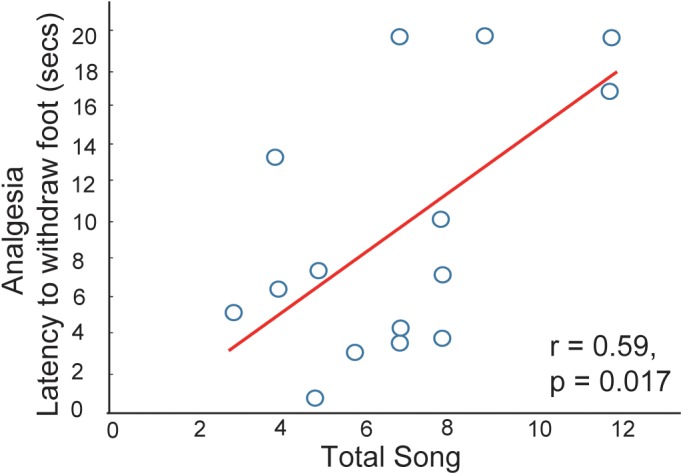
Evidence that vocal-social interactions in starling flocks are associated with pain reduction. Correlation between song production and the latency for a male to withdraw its foot from a hot water bath. Each point represents one individual. Figure redrawn from Kelm-Nelson et al. (2012).

The idea that social interactions in flocks relieve social pain via mechanisms that also regulate physical pain is supported by a song-associated analgesia study in male starlings (Kelm-Nelson et al., 2012). Singing was recorded in gregarious non-breeding flocks or in a mate-directed context. Each bird was then immediately captured and one foot was submerged in a hot water bath (hot enough to be mildly aversive; some birds retracted the foot immediately, but others did not respond). The amount of time it took for each bird to remove its foot from hot water was noted as a measure of analgesia. A linear positive correlation was found between this measure of analgesia and the production of song in flocks, indicating that vocal-social interactions in flocks are associated with pain reduction (Figure 2). A similar relationship was not found between sexually motivated female-directed song and analgesia, which again suggests that mechanisms mediating mate-directed, immediately reinforced behaviors differ from those underlying affiliative behaviors produced in gregarious contexts. This form of analgesia may function to strengthen group cohesion through the reduction of an aversive state. Thus, these findings offer support for the hypothesis that social interactions in non-sexual, gregarious contexts can be negatively reinforced.

Based on the associations revealed by these behavioral studies between singing in flocks and both positive affect and analgesia, we propose that flocking likely involves a combination of incentives and reinforcement mechanisms. This includes positive reinforcement (i.e., a behavior is strengthened because it leads to a positive outcome) and negative reinforcement (i.e., a behavior is strengthened because it leads to reduction of an aversive state). For example, it may be that an aversion to being alone pushes gregarious individuals to join flocks. Joining a flock may thus be negatively reinforced because it reduces the aversive state associated with isolation. Then, once a bird has joined a flock, behaviors produced within the flock, such as singing, may induce a positive affective state that then functions to positively reinforce associations with the flock.

## Mechanisms Underlying Flocking Behavior

### Opioids Modulate the Pain of Being Alone and the Pleasure of Social Contact

Opioid neuropeptides are the neuromodulators that to date have been best studied for their roles in both physical and social pain (Macdonald and Leary, 2005; Eisenberger, 2012). Opioids that bind to mu opioid receptors are also well-known for their rewarding and analgesic properties (Matthes et al., 1996; Trescot et al., 2008; Jhou et al., 2012; Fields and Margolis, 2015). Animals will readily self-administer mu opioid receptor agonists, such as morphine and develop strong CPPs for places associated with mu receptor agonist treatment (Wise, 1989; McBride et al., 1999). Mu receptor agonists also decrease the amount of time rats spend near conspecifics, which has been interpreted to suggest that reward induced by the agonist replaces the need for reward (or relief) that is normally induced by social contact (Herman and Panksepp, 1978; Panksepp et al., 1979). A role for opioids in pain relief induced by social interactions is also supported by past research. For example, reunion of male mice with siblings has been found to induce opioid-dependent analgesia (D’Amato and Pavone, 1993; D’Amato, 1998). Furthermore, the hot water foot dip test of analgesia used in the starling study described above is opioid sensitive (Evrard and Balthazart, 2002; Kelm-Nelson et al., 2012), suggesting that for starlings social contact within flocks (as reflected by flock singing) may release opioids to relieve the pain of isolation.

Data from other species offer more direct support for opioid-mediated social reward and/or reduction of social pain. Mu receptor agonists reduce separation distress vocalizations in rodents and primates; whereas the opioid receptor antagonist naloxone increases these calls (Herman and Panksepp, 1978; Panksepp et al., 1978; Kalin et al., 1988). Distress vocalizations in domestic chicks separated from flock mates were also reduced by systemic treatment with a mu opioid receptor agonist (Warnick et al., 2005). Manipulations of delta and kappa opioid receptors were ineffective, indicating that reduction of social distress induced by social reunion in chicks is mediated selectively by mu receptors. In contrast, peripheral injections of the opioid receptor antagonist naloxone in male zebra finches suppressed “undirected” singing behavior (Dunn and Zann, 1996; Zann, 1996; Khurshid et al., 2010). This is a type of song that is predominantly produced in gregarious flock settings; however, a caveat is that in this study undirected song was produced by birds in isolation rather than in a flock setting. Although differences may be found in birds producing this type of song as part of a gregarious flock, unpublished data in starlings singing in flocks also indicate that mu opioid receptor agonism facilitates singing in gregarious flocks (Riters et al., unpublished data). These findings appear to contradict the studies in rats that show mu agonism decreases time with conspecifics (reviewed above). However, one interpretation is that an optimal level of mu opioid receptor stimulation is needed to facilitate gregariousness, with low doses of MOR agonist stimulating behavior to a point, after which increasing doses suppress behavior (resulting in an inverted-U shaped curve). This idea is supported multiple studies that show inverted-U shaped relationships between mu opioid stimulation and behavior [e.g., for sucrose consumption (Zhang and Kelley, 1997), for self-injection of MOR agonist (Simmons and Self, 2009)]. Thus when taken together, studies to date indicate that opioids that act at mu receptors both reduce vocal behavior indicative of distress and stimulate vocal behavior indicative of a positive (or less negative) affective state, consistent with a role for opioids in the regulation of flocking through both pain reduction (i.e., negative reinforcement) and pleasure (i.e., positive reinforcement).

### A Role for Opioids in the Medial Preoptic Nucleus in Social Interactions in Flocks

There are several brain regions in which the activation of mu opioid receptors can induce reward and analgesia. One such region is the medial preoptic nucleus (mPOA; often referred to as POM in birds) (Tseng et al., 1980; Tseng and Wang, 1992), which has been implicated strongly in the regulation of the affective state associated with birdsong (Riters, 2012; Riters et al., 2017). It is well known across vertebrates that the mPOA stimulates goal-directed, sexually motivated behaviors, including sexually motivated birdsong (Riters and Ball, 1999; Alger and Riters, 2006; Alger et al., 2009). However, this region also appears to play a role in non-sexual vocal-social interactions in flocks of male starlings. In contrast to the inhibition of sexually motivated song by mPOA, lesions of the mPOA tend to promote song in flocks in non-breeding male starlings (Alger and Riters, 2006). In mPOA, opioids that bind to mu receptors inhibit neuronal firing in birds and mammals (Diez-Guerra et al., 1987; Furukawa et al., 1995), suggesting that opioid release associated with vocal-social interactions in flocks may inhibit activity in mPOA to facilitate song in non-breeding flocks.

**FIGURE 3:**
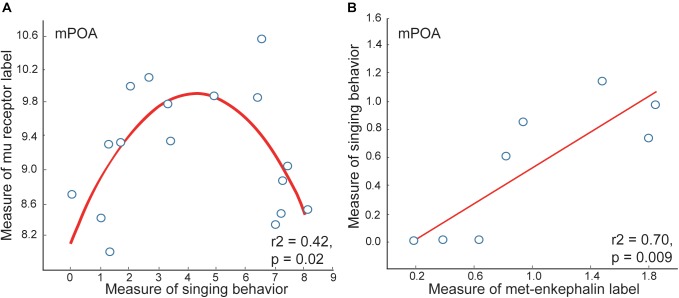
Measures of mu opioid receptor and met-enkephalin immunolabeling in mPOA correlate with vocal-social interactions in starling flocks. **(A)** An inverted-U shaped curve shows the measure of flock song on the *x*-axis and the mean area covered by mu opioid receptor immunolabeling on the *y*-axis. **(B)** A positive linear correlation between flock song on the *y*-axis and the area covered by met-enkephalin immunolabeling. Each point represents one individual. Figures redrawn from Riters et al. (2005) and Kelm-Nelson and Riters (2013).

Opioid measures in mPOA correlate with vocal-social interactions in gregarious flocks. Measures of mu opioid receptor immunolabeling in mPOA correlate positively with vocal-social interactions in gregarious flocks, but only to a point, after which higher rates of song are associated with lower densities of receptor labeling, resulting in inverted-U shaped relationships (Kelm-Nelson and Riters, 2013) (Figure 3). While it could be that opioid signaling decreases in birds singing high rates of non-breeding flock song, met-enkephalin labeling in mPOA relates positively to singing behavior even in the birds singing at the highest rates (Riters et al., 2005). Mu opioid receptors down-regulate in response to sustained occupation by enkephalin (Chang et al., 1982; Harrison et al., 1998), suggesting that high levels of opioid release in mPOA associated with the highest rates of vocal-social interaction may cause mu-opioid receptors to down-regulate (explaining the low mu densities in the second half of the curve; Figure 3).

Opioids (i.e., enkephalins and other mu receptor agonists) in the mPOA, have been shown in rats to induce analgesia as well as reward (analgesia: Tseng et al., 1980; Tseng and Wang, 1992); reward: Agmo and Gomez, 1991, 1993; Le Merrer et al., 2009). These previous data lead to the hypothesis that opioid release in mPOA caused by joining a flock and interacting socially with flock mates underlies both the reduction in pain (i.e., analgesia) and reward (i.e., song-associated CPP) observed in starling flocks. Consistent with this hypothesis, in male starlings, the affective state associated with vocal-social interactions in flocks (measured using CPP) correlated positively with both preproenkephalin (the precursor of the opioid met-enkephalin) and mu opioid receptor mRNA expression levels in mPOA (Riters et al., 2014). Moreover, preliminary data from a study on the mPOA show that selective downregulation of MOR in this region (induced by siRNA infusion) in male starlings suppresses affiliative song and disrupts song-associated reward, (Riters et al., unpublished results).

## The mPOA Accesses Both Canonical Reward and Pain Pathways

**FIGURE 4:**
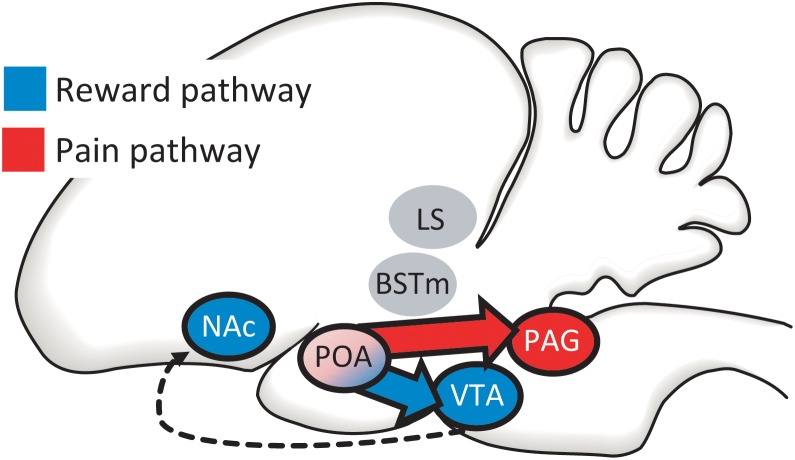
The mPOA directly accesses both canonical reward (in blue) and pain (in red) pathways via projections to (1) the ventral tegmental area [VTA; which then projects to the nucleus accumbens (NAc)] and (2) the periaqueductal gray (PAG). We develop the case that the social pleasure and pain reduction that facilitate and maintain flocking behavior may be modified by these two complementary output pathways from mPOA, with PAG integrating information during social interactions that reduces negative affect and the VTA integrating information leading to social approach and reward. Two additional regions in which opioids are proposed to interact with nonapeptides, the bed nucleus of the stria terminalis (BSTm) and lateral septum (LS) are also shown. The LS, BSTm, POA, PAG, and VTA are all reciprocally connected (connections not shown).

Studies to date highlight opioid activity in mPOA as likely involved in positive and negative reinforcement of song, but this region does not work in isolation. The mPOA receives inputs from multiple brain regions (Chiba and Murata, 1985; Balthazart et al., 1994; Riters and Alger, 2004) and is proposed to integrate information about an individual’s internal state with the external environment so that an animal will produce an appropriate motor output (Wood, 1998; Ball and Balthazart, 2004; Hull and Dominguez, 2006; Alger et al., 2009). Of relevance to mechanisms of positive and negative reinforcement are two key output pathways. (1) The mPOA directly accesses the canonical mesolimbic reward pathway via a direct projection to the ventral tegmental area (VTA) (Balthazart et al., 1994; Riters and Alger, 2004), which sends projections to the nucleus accumbens (NAc) (Husband and Shimizu, 2011) (Figure 4). This has been the best studied pathway for the regulation of motivated, reward-directed behaviors, yet few studies have focused on the role of this pathway in flocking behavior. (2) The mPOA also gains direct access to a well-studied pain pathway via a projection to the periaqueductal gray (PAG; often referred to as the central gray in papers on birds), a region well-known to regulate pain and aversive emotional states (Behbehani, 1995; Macdonald and Leary, 2005; Lieberman and Eisenberger, 2009; Wright and Panksepp, 2011) (Figure 4). This suggests the hypothesis that the mPOA may unite a complementary dual-pathway system to regulate flocking behavior. Below we review data from the few studies involving flocking behavior to date that have examined VTA and PAG. There are certainly other pathways in which opioids modulate pain and pleasure and recent studies identify overlap in circuits regulating pleasure and pain (e.g., Leknes and Tracey, 2008; Mitsi and Zachariou, 2016). We consider the two mPOA output pathways that we highlight here to be a reasonable starting point to begin to reveal mechanisms that reinforce flocking behavior, but additional regions and mechanisms should be considered in future studies.

### A Possible Role for the Mesolimbic Reward Pathway in Flocking Behaviors

**FIGURE 5:**
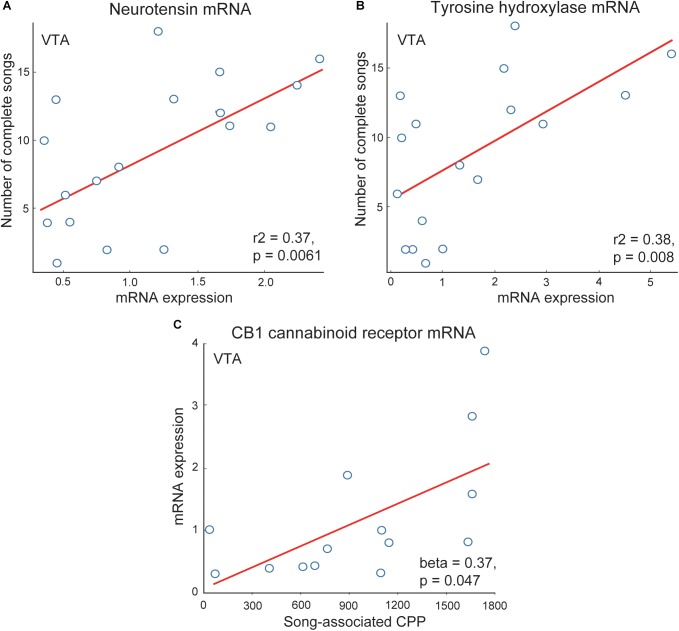
Correlations support roles for neuromodulators in VTA in flocking behavior in starlings. Positive correlations between flock song on the *y*-axis and measures of **(A)** neurotensin and **(B)** tyrosine hydroxylase mRNA in VTA. **(C)** Positive correlation between CB1 cannabinoid receptor mRNA on the *y*-axis and song-associated CPP on the *x*-axis. Each point represents one individual. Figures redrawn from Merullo et al. (2016) and Hahn et al. (2017).

Dense dopaminergic projections from the VTA to the NAc are among the best studied components of the canonical mesolimbic reward pathway [for recent review see (Baik, 2013)]. Multiple studies demonstrate these dopaminergic projections to be crucial for motivated, approach responses to rewarding stimuli (Berridge and Robinson, 1998; Ikemoto and Panksepp, 1999; Ikemoto et al., 2015; Volkow et al., 2017). These projections are highly evolutionarily conserved and underlie multiple motivated, reward directed behaviors, with a few studies suggesting this role may extend to flocking behavior. For example, gregarious species of estrildid finch have more neurons labeled for tyrosine hydroxylase (a rate-limiting enzyme in catecholamine synthesis) in VTA than non-gregarious, territorial species (Goodson et al., 2009a). Tyrosine hydroxylase mRNA in VTA also correlates positively with vocal-social interactions in flocks of male starlings (Merullo et al., 2016) (Figure 5). These studies are consistent with the possibility that dopaminergic VTA projections modulate the motivation to interact with flock mates. Dopamine is also released in the VTA projection region Area X in male zebra finches singing undirected song (a type of song produced commonly in flocks) (Sasaki et al., 2006). Studies also implicate other neuromodulators, including neurotensin and endocannabinoids, in the VTA in non-sexual, vocal-social interactions in starling flocks. Neurotensin strongly modulates activity of dopamine neurons in the VTA (Steinberg et al., 1995; Kortleven et al., 2012; Stuhrman and Roseberry, 2015) and neurotensin mRNA in VTA correlates positively with singing behavior in starling flocks (Merullo et al., 2016) (Figure 5). Endocannabinoid CB_1_ receptors also modulate the firing of dopamine neurons in VTA (Merullo et al., 2016), and CB_1_ receptor mRNA expression in VTA correlates positively with CPP measures of flock song-associated reward (Merullo et al., 2016; Hahn et al., 2017) (Figure 5). In mammals, opioids in the VTA have also been found to indirectly stimulate dopaminergic neurons and motivated approach behaviors, with a similar mechanism identified in songbirds (Gale and Perkel, 2006). However, correlational studies so far do not (Riters et al., 2014), or only weakly (Riters et al., 2005), implicate opioids in the VTA in vocal-social interactions in flocks.

In contrast to the well-studied role of VTA dopamine projections in motivated responses to rewarding stimuli, opioids binding to mu receptors in the NAc are implicated in reward (i.e., the hedonic pleasure induced when a reward is received) (Smith and Berridge, 2007; Berridge, 2009). Although a putative location for the NAc has been identified in birds based on neurochemical and hodological similarity to mammalian NAc (Balint and Csillag, 2007; Balint et al., 2011; Husband and Shimizu, 2011), to date no studies have experimentally examined the role of mu opioid receptors in the NAc in social reward in birds. An important next step in this line of research will be to examine the role of mu receptors in NAc in flocking behavior.

### A Possible Role for a Negative Reinforcement Pathway in Flocking Behaviors

**FIGURE 6:**
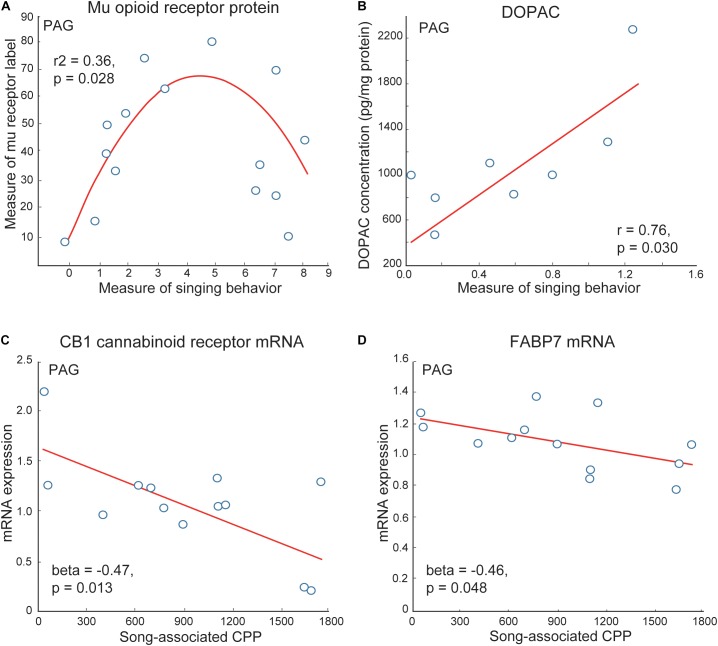
Correlations support roles for neuromodulators in PAG in flocking behavior in starlings. **(A)** An inverted-U shaped curve shows the measure of flock song on the *x*-axis and the mean area covered by mu opioid receptor immunolabeling in PAG on the *y*-axis. **(B)** A positive linear correlation between flock song on the *x*-axis and the dopamine metabolite DOPAC concentrations in PAG on the *y*-axis. Negative correlations between flock song-associated CPP on the *x*-axis and measures of **(C)** CB1 cannabinoid receptor and **(D)** the cannabinoid transporter FABP7 mRNA in PAG. Each point represents one individual. Figures redrawn from Heimovics et al. (2011); Kelm-Nelson and Riters (2013), and Hahn et al. (2017).

The PAG has been well studied as a site in which mu opioid receptors act to induce analgesia (Bodnar et al., 1988; Spinella et al., 1999; Wiedenmayer and Barr, 2000; Morgan et al., 2014). The stimulation of mu opioid receptors in PAG also induces CPP (Olmstead and Franklin, 1997). Studies in birds and mammals suggest that the PAG gathers and integrates information about affective state from other brain regions, including mPOA, which it then relays to vocal production areas so that an animal emits a vocal signal reflective of its emotional state (Jurgens and Pratt, 1979; Absil et al., 2001; Gruber-Dujardin, 2010). In mammals, electrical input from mPOA to PAG stimulates vocalizations produced in positive contexts (i.e., calls produced during sexual behavior in guinea pigs or clucking in monkeys) but not distress vocalizations (i.e., isolation distress calls in guinea pigs or shrieks in monkeys) (Kyuhou and Gemba, 1998; Dujardin and Jurgens, 2006). Data also show that enkephalin opioids and stimulation of mu receptors in the PAG suppress negative vocalizations (i.e., hissing in cats) (Shaikh et al., 1991a,b). In starlings, similar to what was found for mPOA, inverted-U shaped relationships were detected between vocal-social interactions in flocks and measures of mu opioid receptor immunolabeling PAG (Kelm-Nelson and Riters, 2013) (Figure 6). Numbers of neurons labeled for the immediate early gene ZENK (also referred to as Egr-1) and numbers of ZENK labeled neurons double labeled for tyrosine hydroxylase were also higher in PAG in male zebra finches producing undirected song in flocks compared to silent males (Lynch et al., 2008). A similar result was observed in Bengalese finches, *Lonchura domestica* producing undirected song when isolated (Matheson and Sakata, 2015). Studies in male starlings also show that the dopamine metabolite DOPAC in PAG correlates positively with singing behavior in gregarious starling flocks (Heimovics et al., 2011) (Figure 6). Markers for cannabinoids (i.e., cannabinoid receptors and the cannabinoid transporter FABP7) also correlate negatively with song-associated CPP (Hahn et al., 2017) (Figure 6). The function of these relationships must now be tested experimentally using site-specific pharmacological or gene manipulations; however, together with past studies, these correlational data suggest potential roles for the PAG as well as the VTA in vocal-social interactions in flocks and affective state.

The PAG and VTA may modulate motivated behavior by different mechanisms. For example, it has been speculated that infusion of the mu opioid receptor agonist morphine into the PAG induces CPP (Olmstead and Franklin, 1997) by reducing negative affect (rather than by inducing a positive affective state), based on studies showing that a main role for the PAG is to modulate responses to aversive stimuli (Lieberman and Eisenberger, 2009; Wright and Panksepp, 2011). In contrast, morphine in VTA may induce CPP by increasing positive affect, based on studies showing the VTA to be important for reward and approach (Bozarth and Wise, 1981; Wise and Bozarth, 1987; Berridge and Robinson, 1998; Ikemoto and Panksepp, 1999; Ikemoto et al., 2015; Volkow et al., 2017). This suggests that the pleasure and pain that facilitate flocking behavior may be modified by these two complementary output pathways from mPOA, with PAG integrating information during social interactions that leads to pain reduction and the VTA integrating information leading to social approach and reward.

## Integration With Prior Studies on “Nonapeptides” and Flocking Behavior

This review is focused on opioids and the reinforcement of flocking behavior; however, a major focus of current research on group living, including flocking in birds, is on neuropeptides in the vasopressin/oxytocin, “nonapeptide” family (homologs to avian vasotocin/mesotocin) (Goodson, 2008, 2012; Goodson et al., 2009b, 2012a; Goodson and Kingsbury, 2011; Ondrasek et al., 2018; Beery, 2019). Opioids have long been known to alter nonapeptide release (Bicknell and Leng, 1982; Brown et al., 2000), and nonapeptides influence opioid activity to modulate behavioral responses to social and painful stimuli (including the pain of social separation) (Csiffary et al., 1992; Yang et al., 2007, 2011a,b; Moaddab et al., 2015; Amini-Khoei et al., 2017). For example, oxytocin increases tissue sensitivity to opioids (Meguro et al., 2018), and enhances mu opioid receptor agonist induced CPP and analgesia (Moaddab et al., 2015; Meguro et al., 2018). It has been proposed that oxytocin may increase the salience of stimuli that release opioids (Moaddab et al., 2015). This suggests that if vocal-social interactions in flocks release opioids, oxytocin may enhance the reinforcing effects of these social interactions.

Despite the extensive evidence for mechanistic overlap, roles for opioids and nonapeptides in social behavior are not commonly considered together, and it has been suggested that researchers may be ignoring critical, in some cases dominant, input from opioids (Nelson and Panksepp, 1998; Depue and Morrone-Strupinsky, 2005) and overgeneralizing roles for nonapeptides in social behavior (Insel and Shapiro, 1992; McCall and Singer, 2012). Here we provide an overview of studies on nonapeptides and flocking in birds and consider ways in which these peptides may interact with opioids to reinforce flocking behavior.

Like opioids, nonapeptides induce analgesia in mammals (Yang et al., 2007, 2011a,b; Xin et al., 2017). The nonapeptide oxytocin also reduces separation distress vocalizations (Insel and Winslow, 1991; Panksepp, 1992) and induces conditioned social preferences (Kent et al., 2013; Kosaki and Watanabe, 2016). However, these studies also show that oxytocin results in either a modest or no CPP in the absence of a social partner, which may reflect a selective role for nonapeptides in social, not general, reward. In songbirds, nonapeptides are also implicated in positive responses to social, flock-related stimuli. A strong body of research demonstrates that vasotocin-containing neurons in the bed nucleus of the stria terminalis (BSTm) increase gregariousness (i.e., preferences for a large versus a small flock) and reduce anxiety behavior (Kelly et al., 2011). Measures of ZENK in the BSTm in starlings correlate positively with song in gregarious flocks, but not sexually motivated, male song (Heimovics and Riters, 2007). Neurons positive for the immediate early gene c-fos in BSTm that were activated in response to positive social stimuli (i.e., the presence of conspecifics in gregarious finch species) were also found to be vasotocin-positive and proposed to play a role in positively (but not negatively) valenced responses to social stimuli (Goodson and Wang, 2006). The neurons originating in BSTm project to the lateral septum (LS), which is a site in which vasotocin-like receptors promote gregariousness (Kelly et al., 2011). Studies also implicate mesotocin-like receptors in LS in gregariousness (Goodson et al., 2009b, 2012b; Ondrasek et al., 2018), and a study in female zebra finches shows that mesotocin-like receptor antagonist infused directly into LS reduced gregariousness (Goodson et al., 2009b). Together these data provide support for causal roles for nonapeptide projections from BSTm to LS in flocking.

Opioids in the BSTm are also implicated in flocking behavior. In starlings the relationship between vocal-social interactions in flocks and mu opioid receptor labeling in BSTm was curvilinear (i.e., inverted-U shaped), similar to the relationships identified in the mPOA and PAG (Kelm-Nelson and Riters, 2013). This suggests the hypothesis that regions in which nonapeptides and/or opioids modulate gregariousness are part of a network that controls flocking behavior. In support of this, the BSTm and LS (i.e., brain areas in which nonapeptides are implicated in flocking behavior) are reciprocally connected to the mPOA as well as proposed output regions underlying positive and negative reinforcement (i.e., the VTA and PAG) (Riters and Alger, 2004; Goodson, 2005). Electrical input from BSTm to PAG evokes both aversive (i.e., shrieking) and non-aversive (i.e., chattering) vocalizations in monkeys, with injections of the opioid receptor antagonist into PAG reducing the threshold to produce aversive but not, non-aversive calls (Jurgens and Lu, 1993). Met-enkephalin in BSTm also suppresses negative vocal behavior (i.e., hissing) in cats (Brutus et al., 1988). Recent data showing dense concentrations of mesotocin- and vasotocin-like receptors in mPOA in starlings (females in this study) captured in winter when they flock (Ondrasek et al., 2018), suggest that a role for vasotocin in mPOA in seasonal changes in flocking is worth exploring. Studies are now needed to explore roles for opioid/nonapeptide interactions in an expanded neural circuitry that includes mPOA, PAG, VTA, BSTm, and LS.

### A Possible Role for Steroid Hormones in Flocking Behavior

As introduced earlier, many songbirds shift seasonally from pair or solitary living during the breeding season to the formation of flocks after the breeding season. In an early review of flocking behavior, Emlen (1952a) suggested that seasonal increases in steroid hormone concentrations may disrupt a default state of gregariousness by facilitating behaviors disruptive to social cohesion (i.e., agonistic and sexual behaviors) (Emlen, 1952a). Consistent with this idea, in many temperate zone breeding songbirds, gonadal steroid concentrations are low outside the breeding season when birds often congregate in flocks (e.g., Wingfield and Farner, 1978; Dawson, 1983; Van Duyse et al., 2003). Low sex steroid hormones outside the breeding season in male songbirds are associated with the production of relatively shorter and less stereotyped songs (Smith et al., 1995, 1997; Riters et al., 2000; Alger et al., 2016). These songs are less attractive to females and less threatening to male competitors compared to longer, more stereotyped songs that are produced in primary reproductive contexts (Searcy and Yasukawa, 1996; Woolley and Doupe, 2008). Thus, social tolerance may be promoted in flocks through the de-emphasis of song features that induce sexual and agonistic responses.

As concentrations of testosterone in males and estradiol in females rise at the onset of the breeding season, flocks disperse and former flock mates begin to aggressively defend breeding territories and to compete for mates, singing long, stereotyped songs that repel conspecifics and attract mates (Wingfield and Farner, 1978; Dawson, 1983; Smith et al., 1995, 1997; Searcy and Yasukawa, 1996; Riters et al., 2000; Van Duyse et al., 2003; Alger et al., 2016). In some species, treating wintering birds with testosterone increases the frequency of aggressive interactions in flocks and can lead to increased spacing among flock individuals (Emlen and Lorenz, 1942; Baptista et al., 1987; Archawaranon et al., 1991). Furthermore, the mPOA has been identified as a central site in birds and mammals in which testosterone acts to promote agonistic and sexual motivation (Schlinger and Callard, 1989; Watson and Adkins-Regan, 1989a,b; Balthazart et al., 1990; Riters et al., 1998; Coolen and Wood, 1999; Hull et al., 1999; Ball and Balthazart, 2004; Balthazart and Ball, 2007), as well as the production of sexually motivated songs in canaries, *Serinus canaria* (Alward et al., 2013, 2018). Thus testosterone may act in mPOA to dissociate flocks by promoting aggressive and sexual behaviors among flock members.

**FIGURE 7:**
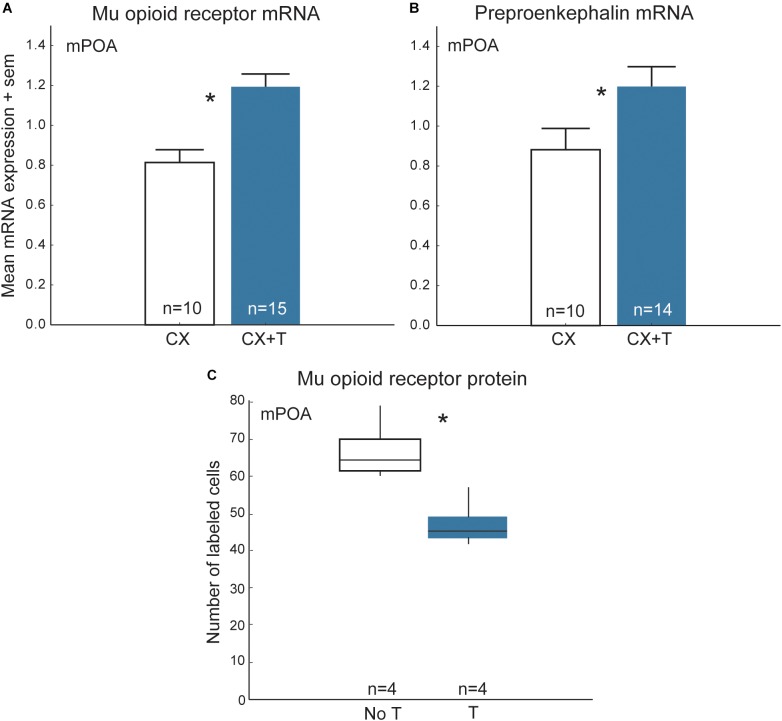
Steroid-dependent changes in opioids in mPOA may modify flocking behavior seasonally in starlings. Mean + sem mRNA expression measures in mPOA for **(A)** mu opioid receptors and **(B)** preproenkephalin in castrated (CX; open bars) and castrated males treated with testosterone (CX + T; filled bars). **(C)** Numbers of cells in mPOA immunolabeled for mu opioid receptor protein in males with naturally low testosterone that were not treated with testosterone (No T) and males that were treated with testosterone (T). The middle lines in the boxes are medians, the outer edges of the boxes are 1st and 3rd quartiles (i.e., the interquartile range). The whiskers represents data within 1.5 ^∗^ the interquartile range (i.e., 1.5 ^∗^ the width of the box). Figures redrawn from Spool et al. (2016, 2018b).

In many vertebrates, opioids in the mPOA and/or their receptors change seasonally and are regulated by steroid hormones (Watson et al., 1986; Hughes et al., 1990; Mateo et al., 1992; Hammer et al., 1994; Eckersell et al., 1998; Holland et al., 1998; Scott et al., 2008; Woods et al., 2010; Spool et al., 2016). [Nonapeptides and/or their receptors also change seasonally and are regulated by steroid hormones, but we limit our discussion here to opioids because they are the main focus of this review and there are several papers that detail region- and species-differences in steroid effects on nonapeptides (e.g., DeVries et al., 1985; Goodson and Bass, 2001; Panzica et al., 2002; Plumari et al., 2004; Goodson and Thompson, 2010)]. One possibility is that steroid-dependent changes in opioids (and nonapeptides) modify flocking seasonally. Results of studies in starlings are consistent with this hypothesis. Treatment of castrated male starlings with testosterone alters opioid markers in a complex fashion, resulting in increased mu-opioid receptor and preproenkephalin mRNA and steroid-related mRNA (i.e., androgen receptors and aromatase) in mPOA relative to controls, but also reducing numbers of immunolabeled mu opioid receptor cell bodies in the rostral portion of mPOA (Figure 7) (Spool et al., 2016, 2018b), a subregion thought to modulate appetitive sexual behavior (Riters and Ball, 1999; Balthazart and Ball, 2007). In female starlings, individuals with elevated concentrations of estradiol tend to have higher preproenkephalin mRNA in mPOA relative to females with low concentrations of estradiol (Spool et al., 2018a). Additionally, in free-living songbirds mu opioid receptor mRNA in the mPOA changes seasonally, peaking during the breeding season (Woods et al., 2010). The results of these studies suggest that seasonal, steroid-dependent changes in opioid activity in mPOA may modulate seasonal changes in gregariousness such as those observed in European starlings.

A study in house sparrows, *Passer domesticus*, another seasonally gregarious songbird offers additional indirect evidence that changes in seasonal flocking behavior are linked to opioid release. In this study testosterone treatment was found to induce analgesia as measured using the opioid-sensitive hot water foot withdrawal test of analgesia described above in starlings (Hau et al., 2004). This suggests that seasonal changes in testosterone may alter opioid release, or tissue sensitivity to opioids, to reduce aversive states induced by solitude at times when it is adaptive for individuals to remove themselves from social flocks. Given the overlap in the role of opioids in both physical and social pain, it may be that a testosterone-induced increase in opioid release or receptor numbers that occurs during the breeding season replaces the need for pain relief that is induced by social interactions in flocks in the non-breeding season.

Although there is much evidence to support a role for steroid hormones in shifting animals from a prosocial, tolerant state to an agonistic, intolerant state, the relationship between steroid hormones and flocking behavior in birds is complex. For example, in many species some level of aggression persists in non-breeding flocks despite low concentrations of circulating steroids, usually as the result of competition over food and roosting resources (Sabine, 1949; Lockie, 1956; Pinxten et al., 2000; Smith et al., 2005), and the flock remains together despite these conflicts. This type of aggression has been found to be regulated by brain site-specific *de novo* steroid synthesis (Heimovics et al., 2015a,b), yet the extent to which local neurosteroid synthesis modulates flocking behavior has not been studied. Furthermore, some seasonally breeding birds mate in colonial settings and exhibit social tolerance even while sex steroid hormones are elevated. Animals in these breeding groups typically defend small spaces around nest sites [e.g., zebra finches (Zann, 1996), magellanic penguins, *Spheniscus magellanicus* (Stokes and Boersma, 2000), black skimmers, *Rynchops niger* (Burger, 1981), cliff swallows, *Petrochelidon pyrrhonota* (Emlen, 1952b)]. Larger colony sizes (i.e., greater numbers of potential competitors) in cliff swallows are associated with greater circulating concentrations of testosterone in both males and females (Smith et al., 2005). Furthermore, in male starlings testosterone was higher in males nesting in a dense colony compared to males nesting at more dispersed sites (Ball and Wingfield, 1987). Thus in many species increases in sex steroid hormones are associated with flock dissociation at the beginning of the breeding season, yet in other species they promote small-scale territoriality and resource guarding within colonial groups without causing flocks to dissociate. Season and species-specific differences in steroid effects on modulators, such as opioids or nonapeptides, in brain regions involved in flocking may reconcile these variable findings across species and seasons.

## Synthesis, Implications, and Conclusion

We propose that studies of songbirds reveal a novel network model for the integration of positive and negative reinforcement processes in non-sexual affiliative social behavior. Most studies on affiliative behavior focus on the positive affective state induced by social contact that rewards individuals interacting together. However, this review highlights that in social animals, affiliative contact is also reinforced because it reduces a negative affective state caused by social exclusion or isolation, thus creating a complementary system (i.e., positive reinforcement from affiliative interactions and negative reinforcement from termination of isolation). In this review we build the case that both of these mechanisms are central to flock formation and maintenance and propose that mu opioid receptor activity in the mPOA may modulate a positive state induced by flocking, via a projection to VTA, and may reduce a negative affective state resulting from social separation, via a projection to PAG. Neural systems that underlie important social behaviors are evolutionarily conserved (Panksepp, 2005, 2016; O’Connell and Hofmann, 2011). This suggests that a molecular/genetic substrate that existed in a common ancestor has been conserved to provide a foundation for the generation of novel social behaviors across vertebrates, and to fine-tune social behaviors to match the ecological needs of individual species. Thus studies of songbird flocking may advance the understanding of how the molecular substrates underlying social reinforcement have evolved across vertebrates, including humans.

In humans, profound deficits in gregarious social interactions (i.e., playful, non-sexual social interactions) are associated with mental disorders, including depression and autism spectrum disorders. Although several animal models can be used to study goal-directed (i.e., mate- or rival-directed) behaviors, songbirds are one of the only experimental systems to model aspects of learned vocal communication in a non-sexual, yet affiliative context in adults. A survey of studies on humans reveal parallels to songbird studies. For example, in human studies vocal behaviors [i.e., undirected swearing, affiliative social laughter, and vocal repetition or rhythmic respiration (e.g., during meditation)] induce analgesia and/or are associated with a feeling of well-being (Stephens et al., 2009; Dunbar et al., 2012; Ahmed et al., 2014), similar to what has been observed for vocal behaviors produced in flocks in songbirds. Furthermore, in humans both reward and pain neural networks are implicated in social reward and the pain of social rejection (Macdonald and Leary, 2005; Lieberman and Eisenberger, 2009; Eisenberger, 2012), similar to evidence we review here in songbirds. Studies of flocking behavior in birds thus have the potential to provide insight into mechanisms by which behaviors that involve vocal-motor-respiratory stimulation (e.g., controlled breathing, ohms during meditation, swearing and laughter) may naturally promote opioid or nonapeptide release and positive (or less negative) affect in humans. Information provided by songbirds about basic mechanisms underlying affiliative social interactions may also provide important insights into mechanisms underlying positive social interactions that are disrupted by mental illness in humans.

## Author Contributions

LR, CK-N, and JS wrote, edited, and revised the manuscript.

## Conflict of Interest Statement

The authors declare that the research was conducted in the absence of any commercial or financial relationships that could be construed as a potential conflict of interest.
